# Better educational signage could reduce disturbance of resting dolphins

**DOI:** 10.1371/journal.pone.0248732

**Published:** 2021-04-02

**Authors:** Roarke E. Donnelly, Alex Prots, Christl A. Donnelly

**Affiliations:** 1 Biology Program, Oglethorpe University, Atlanta, GA, United States of America; 2 Department of Statistics, University of Oxford, Oxford, United Kingdom; 3 MRC Centre for Global Infectious Disease Analysis, Department of Infectious Disease Epidemiology, Imperial College London, London, United Kingdom; Wildlife Conservation Society Canada, CANADA

## Abstract

Spinner dolphins on Hawai‘i Island’s west coast (*Stenella longirostris longirostris*) rest by day in protected bays that are increasingly popular for recreation. Because more frequent interactions of people with these dolphins is likely to reduce rest for dolphins and to explain recent decline in dolphin abundance, the National Oceanic and Atmospheric Administration (NOAA) proposed stricter rules regarding interactions with spinner dolphins near the main Hawaiian Islands and plans to increase enforcement. Simultaneous investment in public education about both interaction rules and their biological rationale has been and is likely to be relatively low. To test the hypothesis that more educational signage will reduce human-generated disturbance of dolphins, a paper questionnaire was distributed to 351 land-based, mostly unguided visitors at three dolphin resting bays on Hawai‘i Island’s west coast. Responses indicated that visitors wanted to see dolphins, were ignorant of interaction rules, were likely to read signs explaining rules and their biological rationales, and were likely to follow known rules. Therefore, investment in effective educational signage at dolphin resting bays is recommended as one way to support conservation of spinner dolphins on Hawai‘i Island’s west coast and similar sites in the Hawaiian archipelago.

## Introduction

Wildlife tourism is a large and rapidly growing sector of the worldwide tourism industry and has great potential to affect biodiversity conservation [1, 2]. As the touring public has more contact with biodiversity and becomes more familiar with its threats, this portion of the public is more likely to facilitate biodiversity conservation with pro-environmental behaviors and with support for relevant legislation and funding [3–5]. However, increased human interaction with wildlife can also reduce fitness and abundance of target species. For example, mountain gorilla (*Gorilla beringei beringei*) mortality increased with transmission of respiratory pathogens from tourists and/or guides, marine iguana (*Amblyrhynchus cristatus*) immunological capabilities decreased with an increase in tourism intensity, and bottlenose dolphin (*Tursiops sp*.) abundance decreased with an increase in tour boat visitation [6–8]. To reduce these impacts of tourism on wildlife, management agencies often draft and administer policies that use a combination of policy instruments, such as regulations, incentives, and education [9, 10]. It is particularly important that a policy has an effective mix of instruments when the focal wildlife is predisposed to tourism impact. Spinner dolphins on the west coast of Hawai‘i Island (*Stenella longirostris longirostris*) exhibit this predisposition.

Spinner dolphins on Hawai‘i Island’s west coast are genetically distinct from other populations in the archipelago [11] and exhibit a rigid daily behavior pattern that makes them especially vulnerable to human recreational activity. They rest in sand-bottomed, protected bays during the day and cooperatively forage in deep water near the resting bays at night [12]. The preferred daytime habitat is rare [13] and easily accessed by guided watercraft tours and by the unguided public via water and—in most cases—land. Over the last 20–30 years, more wildlife tourism in Hawai‘i [14, 15] has translated to more visits by people to bays, more people in the bays [16], dolphins spending most of their day in close proximity to swimmers and boats [17, 18], and less time for dolphins to rest [18, 19]. Because these trends have coincided with a decrease in abundance of spinner dolphins on Hawai‘i Island’s west coast [20], some believe wildlife tourism is a meaningful threat to spinners and justifies tighter regulation of human interaction with dolphins [18, 19].

As with all marine mammals in US waters, the spinner dolphins in the Hawaiian Islands are protected by the Marine Mammal Protection Act. However, the rule proscribing level b harassment (i.e., disturbance) of dolphins can be difficult to enforce in the water and in court, like similar rules regarding dolphin interactions in other countries [21]. For these reasons, NOAA has repeatedly announced since 2005 that it is considering several new rules to reduce disturbance of spinner dolphins within 2 nautical miles of the main Hawaiian Islands. The proposed rules included barring people from moving within 46m or 91m of dolphins (swim-with and 50 or 100yd approach rule) and from entering known dolphin resting bays for part of each day (time area closure rule) [22, 23]. The swim-with and 50yd approach rule is the preferred alternative in the draft Environmental Impact Statement [19] and was the central focus of recent public information sessions and a recent public comment period. These efforts suggest NOAA is heavily investing resources in regulation of interactions with dolphins and enforcement thereof.

Resource investment in education as a complementary strategy for conservation of spinner dolphins in the Hawaiian Islands appears much more limited, assuming that investment is positively correlated with quantity and effectiveness of work product. At present, the agency teaches the public about legal interaction with dolphins using the federal website, tour operators certified as Dolphin SMART (“T” stands for teaching), and educational signs at bays [24]. Available evidence suggests that at least the latter two strategies appear underfunded and/or ineffective. Only six of the many tour operators in the Hawaiian archipelago and none operating on Hawai‘i Island are certified as Dolphin SMART [25]. Similarly, existing educational signs at most dolphin resting bays [26, see sign titled Don’t Feed Wild Dolphin] fail to follow best practice principles. Because they are few, small in area, and out of most sightlines (pers. obs. on Hawai‘i Island’s west coast by RD and AP), they do not conform to the practice of making signs obvious to the target audience [27–29]. Because the signs lack a reasonable detailed rationale for the law they describe, they are less likely to incentivize compliance with the law [30, 31]. Given deviation of existing signage from best practice principles and that signs and education in general have had variable [27, 32] but occasionally substantial positive effect on behavior of the target audience [27, 30], we think more effective signage might be able to make a meaningful contribution to conservation of spinner dolphins in the Hawaiian Islands. More specifically, we believe such signs could reduce proscribed behavior [30, 31, 33, 34], be more cost effective than enforcement of regulations [35, 36], and dramatically reduce litigation costs by making it clear that those who violated the law had ample opportunity to learn about it.

Here, we test the hypothesis that more obvious and detailed educational signage will reduce the rate at which spinner dolphins in Hawai‘i will be disturbed with a survey of land-based visitors at three dolphin resting bays on Hawai‘i Island’s west coast. More specifically, we use the responses to a paper questionnaire to assess four predictions regarding bay visitation: (a) most people visit—at least in part—to see dolphins; (b) most visitors are ignorant of law regulating human-dolphin interactions and its biological justification; (c) most visitors are likely to read encountered signs explaining legal limitation of human-dolphin interactions; and (d) most visitors are likely to follow the law once they known about it.

## Methods

### Study permission

This study was reviewed and approved in writing by the Oglethorpe University Institutional Review Board for Research with Human Participants. The Board did not issue an approval number. This study was also reviewed and approved in writing by the relevant landowners and local non-profit organizations: Friends of Ho‘okena Beach Park, Hawai‘i County, Hawai‘i Division of Aquatic Resources, Kama‘āina United to Protect the ‘Āina, Keōua Hōnaunau Canoe Club).

### Field sites

**Fig 1:**
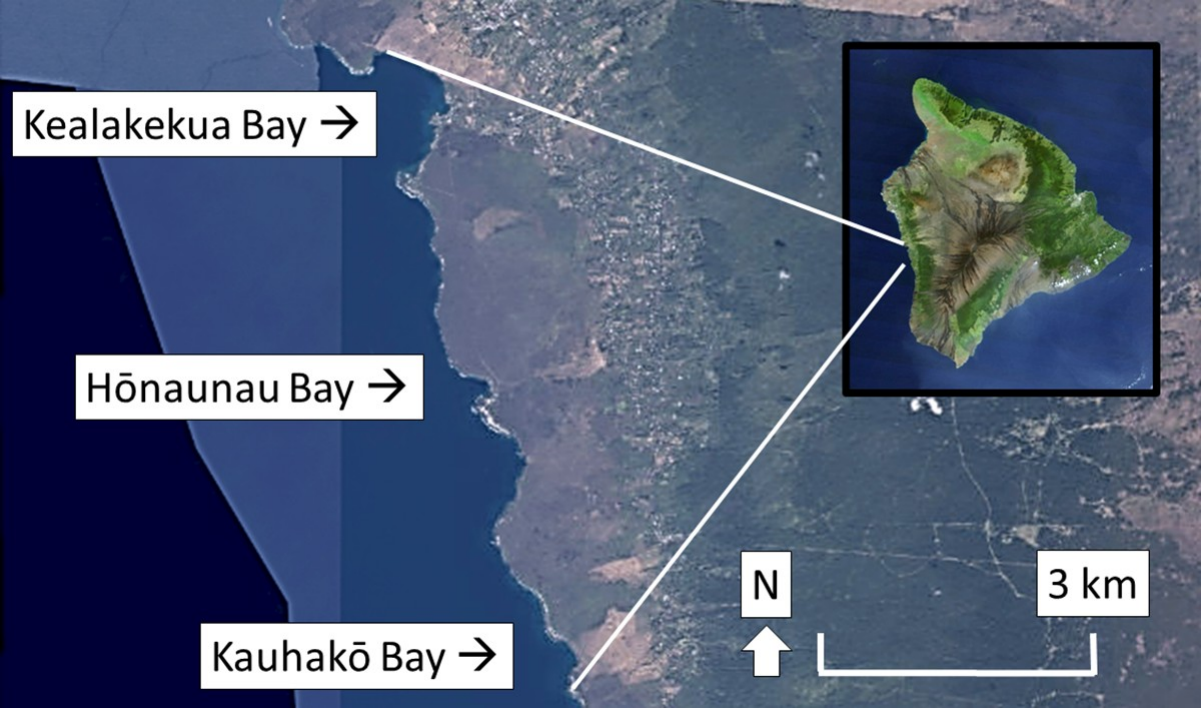
Field sites. We conducted our survey at three dolphin resting bays on the west coast of Hawai‘i Island (inset) in the central Pacific Ocean. Map data: USGS National Map, Orthoimagery, October 2020.

We conducted our survey at the three bay on Hawai‘i Island’s west coast that were frequented by spinner dolphins [18], that were accessible to the public by both land and water, and that would be affected by the time area closure discussed in the draft Environmental Impact Statement on dolphin interactions [19]; these bays are named Kealakekua, Hōnaunau, and Kauhakō (Fig 1). Although the bays were no more than 12km apart, they varied in live coral cover, distance between parking and bay’s shore, recreational amenities, and—most importantly for this study—existing educational signage.

**Fig 2:**
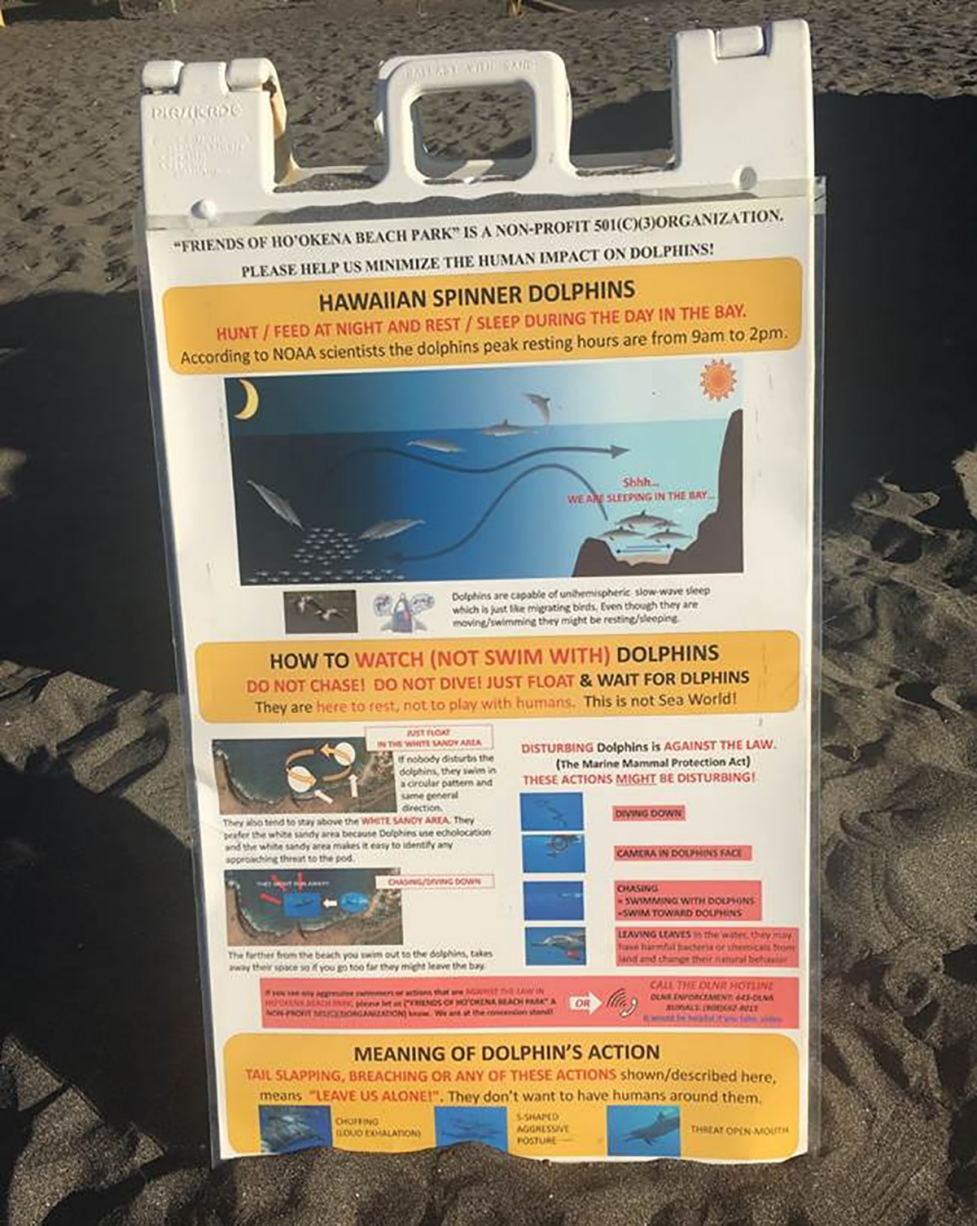
Type of sign found only at Kauhakō Bay. This type was more numerous, larger in area, closer to routes most visitors used to access the bay, and more detailed than the type found at Kealakekua and Hōnaunau Bays [26]. Republished from a photograph by Sam Katz under a CC BY license, with permission from Harlan Miyoshi, original copyright 2017.

Kealakekua and Hōnaunau Bays both had high live coral cover and very limited recreational amenities and signage, but differed in parking distance. Visitors accessed land at Ka‘awaloa on the north end of Kealakekua via a steep and rocky 3.2km hike, a 2km paddle (one-way distances) guided by one of three kayak tour operators who were permitted to land, and—rarely—a 2km swim. Guided paddlers appeared to compose less than one third of the visitors at Ka‘awaloa (pers. obs. RD and AP). Guides and their clients were not allowed to land at the other two bays and, thus, did not participate in our survey. Visitors accessed land at Pae‘a on Hōnaunau Bay via a <0.2km walk on a flat road. Both bays had a few (two or three) signs with information on regulation of human-dolphin interactions that were generally small relative to signs at Ho‘okena Beach Park on Kauhakō Bay (~0.14m^2^), limited to description of penalties for and vague harm/risk from disturbing marine mammals [26], and/or out of the sightlines of most visitors. Hōnaunau and Kauhakō were comparable in terms of parking distance, but differed in live coral cover, recreational amenities, and educational signage. Kauhakō had much less live coral cover and more amenities (e.g., large sandy beach, concession stand, campsites). It also had signs that were more abundant (six) and that tended to be larger in area (~0.42m^2^), to be in the sightlines of more visitors, to have more information on biological justification for regulation of human-dolphin interactions, and to be funded and maintained by a member of a local community organizations (Fig 2). We expected visitor demographics such as age and visit frequency to co-vary with bay characteristics (prediction e).

### Survey

We developed a questionnaire (S1 File) in English that was composed of a brief rationale for the survey, a list of the institutions that granted permission to conduct the survey, the principal investigator’s name and contact information, and 24 mostly closed-ended questions about visitor demography, knowledge, and opinions. The introduction was written in collaboration with a specialist in Human Dimensions of Wildlife at NOAA. The formats and contents of questions were written to reflect best practices [37] and to elicit the targeted information, respectively. To assess our success at eliciting targeted information and to direct editing of the questionnaire before collecting data in the field, we assessed answers from eight undergraduate students and staff at Oglethorpe University. We did not offer the questionnaire in languages other than English because our experience with the study sites and conversations with people living near the study sites indicated that the vast majority of bay visitors spoke English.

We distributed the questionnaire to visitors at one of the three bays per day for a total of 7 days between 5/16/19 and 5/23/19. We controlled for potential effects of day of week on visitor type and number by visiting bays on a repeating 3-day cycle (Hōnaunau, Kealakekua, Kauhakō, repeat). On each survey day, we distributed and collected questionnaires from 7:45AM to 2:15PM and attempted to collect 65 surveys. Sixty-five was the maximum number we thought we could collect each day at the bay with the lowest visitation rate and corresponded to a margin of error of less than 12.5% for proportions estimated with 95% confidence, assuming a large population size. On two days when we collected our target number of questionnaires by ~12:30PM, we stopped surveying earlier than usual. After twice surveying visitors at each bay, we surveyed visitors at Kealakekua Bay on a third day to bring that bay’s sample size closer to that of the other bays.

Once at a bay for a survey, we (i.e., the three authors) asked all visitors for informed consent (verbal) and to complete a paper questionnaire if they were within view, were on land, were > 15yrs old, had not previously participated in the survey, and stayed at the bay >10min. For respondents who were less than 18yrs old, we obtained verbal consent for participation from parents or guardians. RD trained AP and CAD to make the request with a short greeting, description of the survey topic based on the questionnaire’s title, and an estimate of the time required to complete the questionnaire. If a potential respondent repeatedly assisted other visitors with kayaking or snorkeling gear, we asked if they were a guide and noted which questionnaires were completed by guides. If we were unsure if a visitor met the age requirement, we asked about their age. We offered a granola bar in exchange for a self-completed questionnaire. We also noted the number of visitors that declined to participate in the survey, the number of questionnaires that we distributed but were not returned, and the sum of these numbers (functional rejections) so that we could calculate the response rate:

$$100*\frac{collected\;questionnaires}{collected\;questionnaires+funcational\;rejections}.$$

In four instances, we read questions to and recorded answers from visitors who could not read without glasses or write due to an illness.

At the end of the first survey cycle through the bays, females had completed considerably more questionnaires than males. To assess the cause of this inequity, we recorded number and apparent gender of visitors that declined to complete the questionnaire during the second cycle through the bays. We also assessed the number and apparent gender of all visitors on land and over 15yrs of age at Kauhakō and Hōnaunau Bays with a 20-minute visual search after completion of the morning survey on 5/23/19.

### Statistical analysis

We assessed the degree to which samples of bay visitors were sufficiently representative of the bay visitor population in several steps. First, we formally (i.e., with statistical hypothesis tests) compared bay-specific response rates, gender ratios, and representation of native Hawaiians among collected questionnaires with χ^2^ Tests of Homogeneity and, if appropriate, orthogonal contrasts. We considered Hawaiian ancestry because Hawaiians are indigenous in the islands and all field sites are on or next to properties that were managed to preserve traditional Hawaiian culture. If there was no effect of bay on response rate, gender ratio, or representation of native Hawaiians and the expectation was to exceed a threshold, we informally (i.e., without statistical hypothesis tests) compared the aggregate value to the threshold. If there was no effect of bay on any of these variables and there was an exact quantitative expectation, we pooled the data across bays and compared the aggregate value to expectation with a χ^2^ Goodness of Fit Test. If there was a bay effect on any of these variables, we informally compared the mean values for the bays to expectation. We did not consider other visitor demographics (e.g., age) with respect to sample representation, because we could not reasonably generate quantitative expectations. For response rate, we expected a minimum value of 75% of eligible visitors [38]. For gender ratio, we expected 0.96 females per male (15-50yrs of age) [39]. For representation of native Hawaiians, we expected 18% of the sample; we generated this expectation by reducing the percent of Hawai‘i County that was native Hawaiian or other Pacific islander (35%) [40] to account for contribution of other Pacific islanders and reasonable dilution by non-Hawaiian tourists. Lastly, we also informally compared the respondents’ gender ratio to that ratio from our visual survey of visitors at Hōnaunau and Kauhakō and from visitors who declined our invitation to complete a questionnaire.

We also assessed the degree to which the 15 questions most relevant to this study (Table 1) supported predictions a-d related to our research hypothesis (see end of Introduction) and prediction e related to analytical methods (see end of Field sites). The details of this assessment depended on attributes of the focal question. If the question yielded a categorical variable and allowed simultaneous selection of multiple choices (Type 1 questions; #1, 10, 13, and 14), we first assessed independence of choices with a χ^2^ Test of Independence. Choices that were rarely selected and violated the assumption that <20% of matrix cells had expected counts <5 were dropped from further analysis. Dependent choices were assembled into a group from which we retained only the choice with the most selections. We then tested for differences in the distribution of selected choices across bays using a χ^2^ Test of Homogeneity and, when appropriate, orthogonal contrasts.

**Table 1:** Questions for which responses were analyzed. See S1 File for all questions and related skip logic.

Number	Text
1	What did you plan to do while visiting this bay? Please check all options that apply.
2	When you planned today’s visit to this bay, how interested were you in seeing each of the animal types in the table below?
7	To what extent do you agree or disagree with the following statement? *If I see a sign in a park that has information on a local natural resource*, *then I read the sign*.
10	Spinner dolphins frequently visit this bay during the day. What attracts them to this location? Please check all options that apply.
11	Is human interaction with spinner dolphins in this bay currently limited by law?
13	What interactions of humans with spinner dolphins are currently illegal within 2 nautical miles of shore on main Hawaiian Islands? Please check all options that apply.
14	From where have you learned about existing law limiting interactions of humans with spinner dolphins? Please check all options that apply.
15	To what extent would you agree or disagree with a law—if it existed—that attempted to protect Hawaiian spinner dolphins by making it illegal for humans to intentionally move by any means within 50yards (equals 150feet & 46meters) of dolphins?
16	To what extent would you agree or disagree with a law—if it existed—that attempted to protect Hawaiian spinner dolphins by closing portions of three West Hawai‘i bays to people from 6am-3pm each day?
18	Including your current visit, how many times have you visited this bay in the last year?
20	Are you a U.S. citizen?
21	Are you a resident of the state of Hawai‘i? That is, do you spend the majority of each year in Hawai‘i at property that is owned by you or your immediate family?
22	Do you consider yourself to be native Hawaiian by blood/ancestry?
23	What is your age in years?
24	What is your gender?

We analyzed responses to questions that yielded a categorical variable and that allowed selection of only one choice (Type 2 questions; #11, 20, 21, 22, and 24) like responses to Type 1 questions, but beginning with a χ^2^ Test of Homogeneity across bays. We excluded categories from analysis if they violated the assumption that <20% of matrix cells had expected counts <5. If there was no bay effect and we could obtain an expected count, we pooled data across bays and applied a χ^2^ Goodness of Fit Test. We based expected counts on equal probability (#11), state census (#21, see first paragraph in this section), and a global dataset (#24, see first paragraph in this section). For #11, we interpreted selection of “unsure” as a lack of knowledge and scored it as incorrect.

We analyzed responses to focal questions that each yielded a numerical variable (Type 3 questions; #2, 7, 15, 16, 18, and 23) by testing for an effect of bay with a Kruskal-Wallis test. If there was an effect, we identified pairs of bays that differed with a Dunn’s test and Bonferroni adjustment. For question #2, we scored “not interested”, “interested”, and “very interested” as 0, 1, and 2, respectively. For questions #7, 15, and 16, we scored “strongly disagree”, “disagree”, “unsure”, “agree”, and “strongly agree” as integers 1, 2, 3, 4, and 5, respectively.

We used formulas in Microsoft Excel (2016) to run all χ^2^ and related post hoc tests, with the conservative Yates Correction for Continuity, if appropriate. We used SPSS v.25 (2017) to perform all other tests. For all statistical hypothesis tests, we set α at 0.05.

## Results

### Sample quality and prediction e (bay effect)

We collected 351 questionnaires that were at least partially completed. Four of these questionnaires were completed by guides of kayak tours at Kealakekua and do not contribute to analysis unless noted. The remaining 347 collected questionnaires were fairly equally distributed across bays (Kealakekua = 113, Hōnaunau = 129, Kauhakō = 105). Of the three variables used to assess sample representation, only one varied with bay identity; native Hawaiians were least and most common at Kealakekua and Kauhakō, respectively, and seemed under-represented at all bays (Table 2). Aggregate response rate (80% of 439 requests) and aggregate gender ratio (Table 2; all respondents selected male or female from available choices) were greater than the minimum threshold for acceptability and expectation, respectively. However, the aggregate gender ratio from the paper questionnaire (1.5, n = 335) appeared to be similar to the gender ratio from the independent visual survey at two bays (1.3, n = 150) and there was little difference between rejection rates for the genders (16 females and 18 males during the second cycle through the bays). Other respondent demographics also varied among bays; rate of bay visitation, US citizenship, Hawai‘i residency, native Hawaiian ancestry, and age were least and greatest at Kealakekua and Kauhakō, respectively (Table 2).

**Table 2:** Demography of questionnaire respondents. For all categorical characteristics except gender, there was a bay effect (χ^2^ > 7.8, df = 2, P < 0.02; Table 1: Q20-22) and different superscript letters within a row indicate differences between bays (χ^2^ > 4.3, df = 1, P < 0.05). For gender, the asterisk indicates that the aggregate statistic differed from expectation (χ^2^ > 16.4, df = 1, P < 0.001; Table 1: Q24). For age and visitation rate there was a bay effect (H > 28.0, n > 326, P < 0.001; Table 1: Q18, 23) and different superscript letters within a row indicate differences between bays (|Z| > 4.0 and P < 0.001).

	Bay(s)			
Respondent characteristic	Kealakekua	Hōnaunau	Kauhakō	All
US citizenship (% / n)	70.3 / 111 ^a^	82.5 / 126 ^b^	92.1 / 101 ^c^	81.6 / 337
Hawai’i residency (% / n)	6.6 / 76 ^a^	20.4 / 98 ^b^	50.0 / 92 ^c^	26.7 / 266
Native Hawaiian ancestry (% / n)	0.0 / 102 ^a^	1.8 / 114 ^a^	6.0 / 99 ^b^	2.5 / 315
Gender (females per male)	1.4	2.0	1.2	1.5*
Visitation rate (mean number during last year ± SE)	2.0 ± 0.9 ^a^	9.0 ± 2.8 ^b^	20.4 ± 6.4 ^c^	10.0 ± 2.2
Age (mean years ± SE)	36.9 ± 1.5 ^a^	46.1 ± 1.6 ^b^	48.4 ± 1.5 ^b^	43.8 ± 0.9

### Prediction a: Interest in dolphins

**Fig 3:**
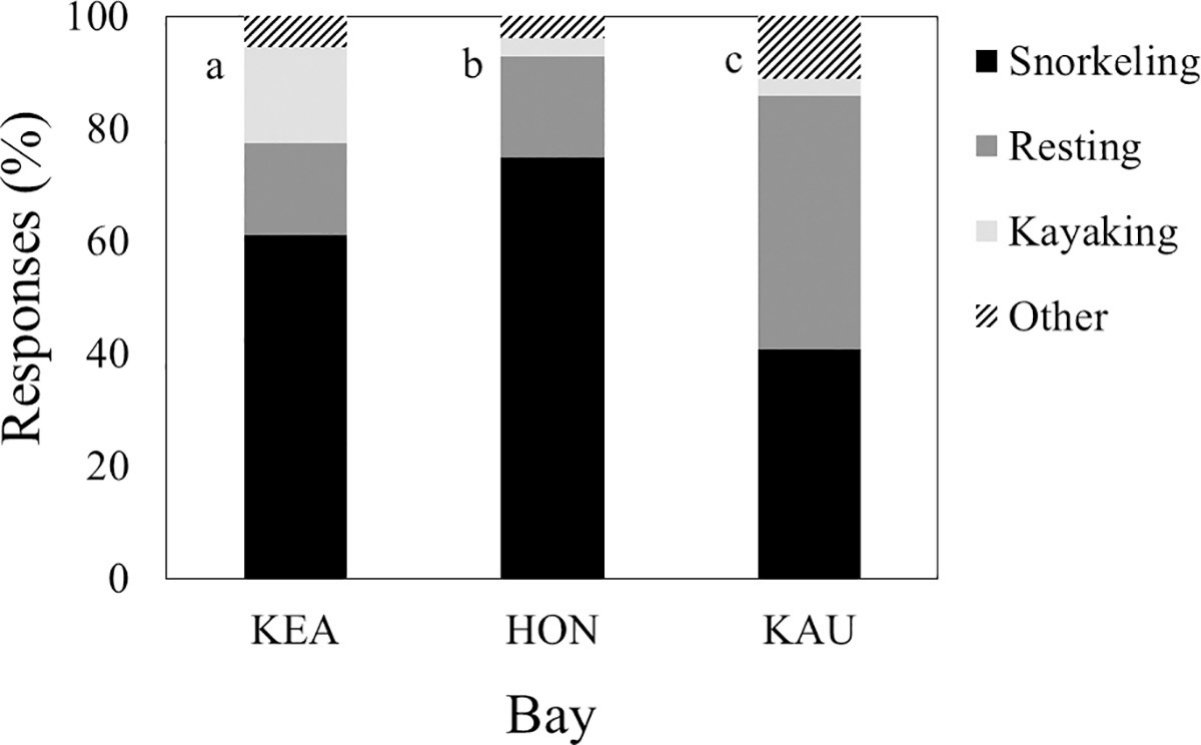
Activities in which respondents planned to engage. Sample sizes varied: Kealakekua = 164, Hōnaunau = 154, and Kauhakō = 133. Snorkeling significantly and positively correlated with swimming, viewing scenery, and watching animals. Resting significantly and positively correlated with camping, fishing, and tanning. Different letters by bars indicate significantly different distributions of planned activities.

**Fig 4:**
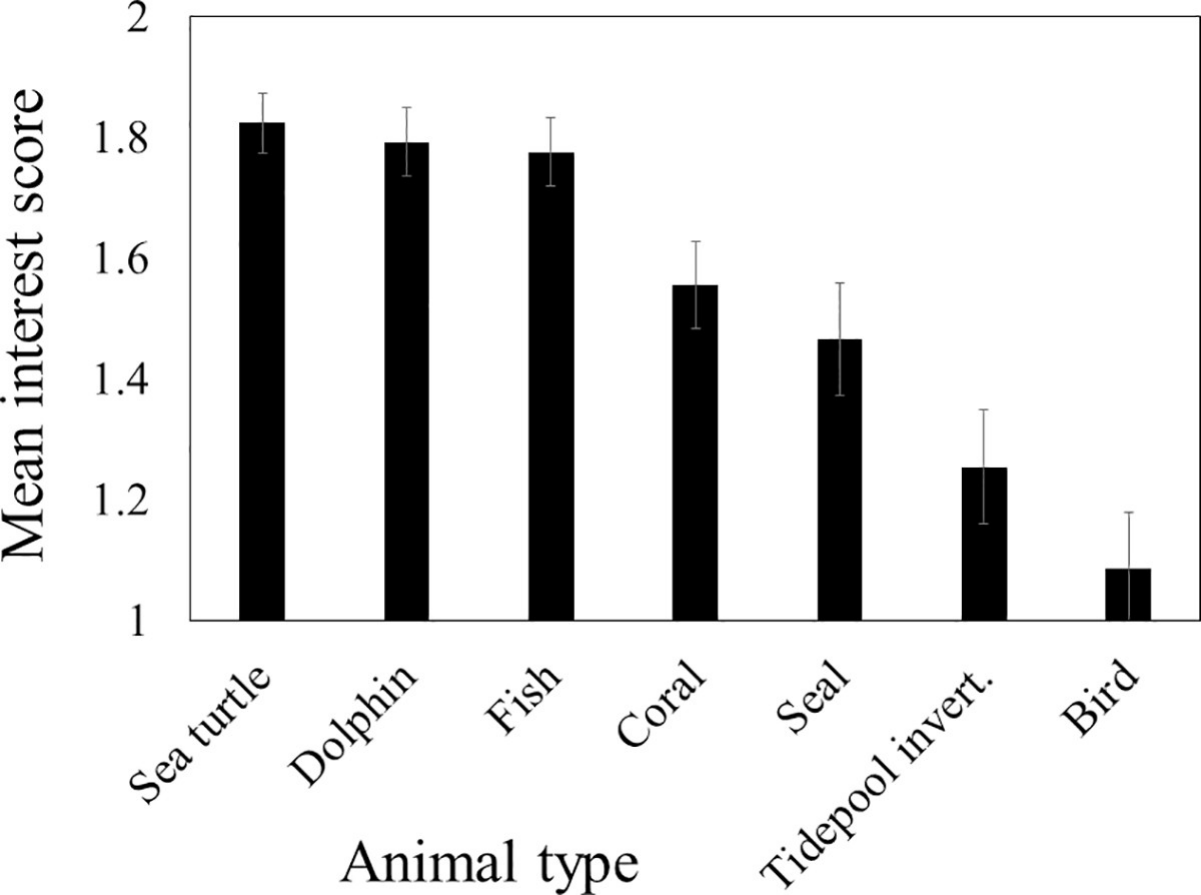
Level of interest in seeing animal types. We translated responses of “not interested”, “interested”, and “very interested” in seeing the animal type to interest scores of 0, 1, and 2, respectively. Sample size for each animal type ranged from 204 to 236. Error bars depict the 95% confidence intervals.

Respondents reported a different distribution of planned activities at all bays (Fig 3; χ^2^ = 77.1, df = 6, P < 0.001; for both contrasts χ^2^ > 20, df = 3, P < 0.001; Table 1: Q1). However, snorkeling and its positive correlates (watching animals, swimming, and viewing scenery) were the most common planned activities at Kealakekua and Hōnaunau and the second most common planned activities at Kauhakō. Resting and its positive correlates (camping, fishing, and tanning) were the most common planned activities at Kauhakō. For those respondents planning to watch animals, interest in each type of animal was uniform across all bays (for all tests: H < 6.0, df = 2, P > 0.05; Table 1: Q2) and greatest for sea turtles, dolphins, and fish (Fig 4).

### Prediction b: Knowledge of law

**Fig 5:**
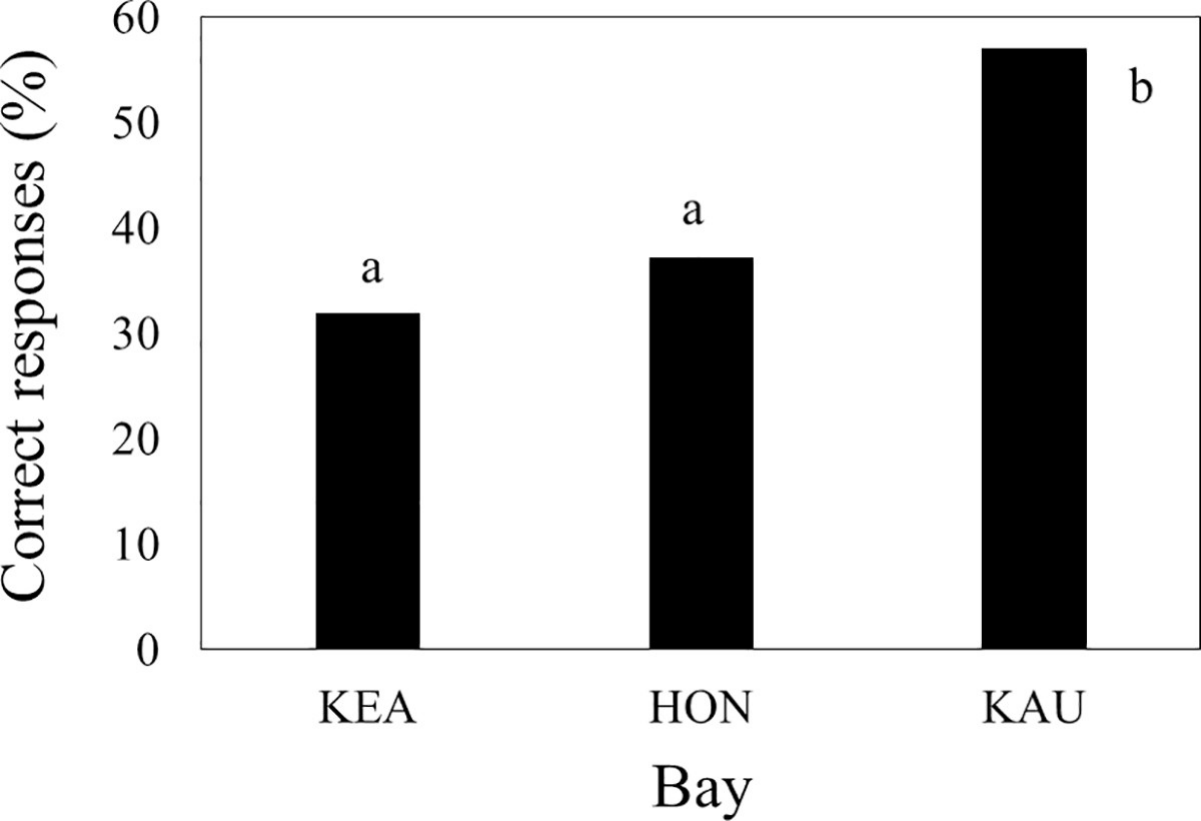
Knowledge that existing law limited interactions of people with dolphins in the studied bays. Sample sizes varied: Kealakekua = 113, Hōnaunau = 129, Kauhakō = 102. Different letters by bars indicate significantly different knowledge.

**Fig 6:**
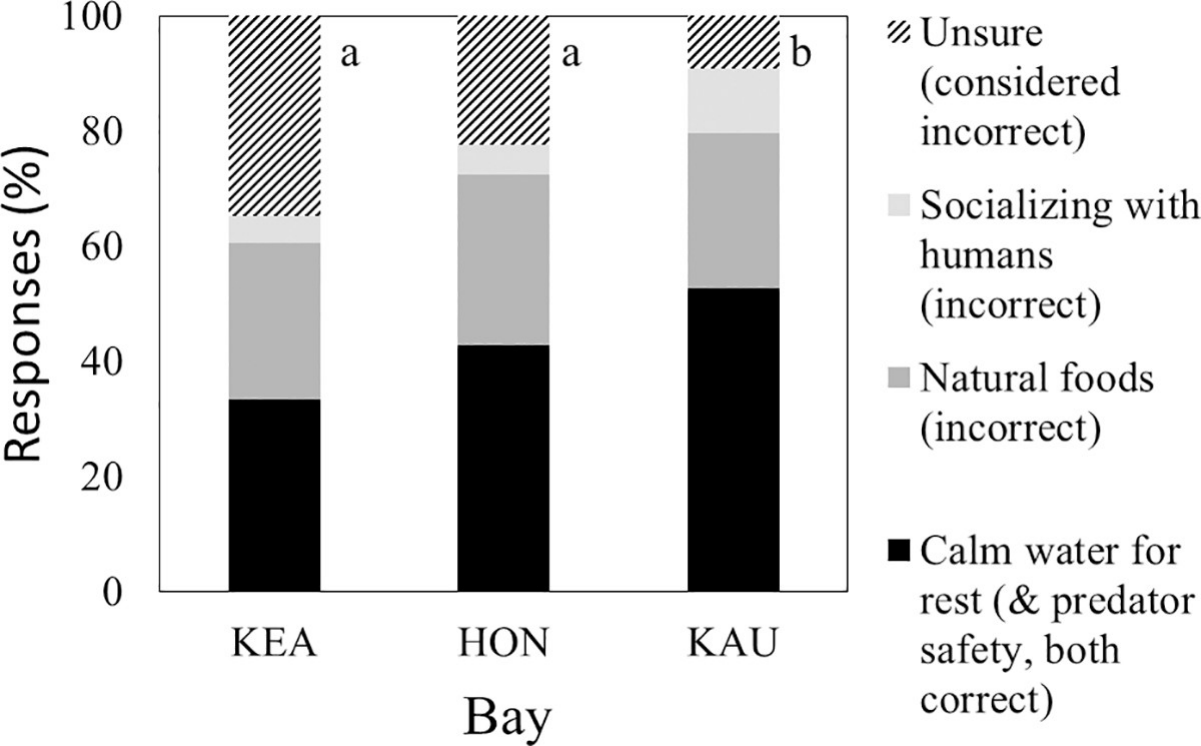
Explanations for presence of dolphins in bays. Sample sizes varied: Kealakekua = 144, Hōnaunau = 178, Kauhakō = 152. Different letters by bars indicate significantly different distributions of explanations.

Knowledge of current legal limits on interactions of people with spinner dolphins varied among bays (χ^2^ = 15.2, df = 2, P < 0.001; Table 1: Q11), with fewer visitors knowing of such limitations at Kealakekua and Hōnaunau than at Kauhakō (Fig 5; χ^2^ = 14.5, df = 1, P <0.001). When respondents who knew of legal limitation on interactions were asked to identify illegal interactions from a list of four options, 21.9% (n = 142) answered “unsure” and the remainder tended to select all choices other than “unsure” (Table 1: Q13). The high positive correlations among answers other than “unsure” (all χ^2^ > 39.9, df = 1, P <0.001) prevented formal statistical testing for equal distribution of selections at bays. However, it also suggested that respondents who knew at least some interactions were illegal and who thought they understood the law usually selected all available choices, including two that were not inherently illegal (i.e., intentionally moving within 50yd of a dolphin by swimming and intentionally moving within 50yd of a dolphin by watercraft with motor in gear). The four respondents who were kayak guides also selected all available choices other than “unsure”. Knowledge of the dolphin biology that explains current legal limits on interactions of people with dolphins also varied among bays (χ^2^ = 34.4, df = 6, P < 0.001; Table 1: Q10), with fewer visitors reporting correct answers at Kealakekua and Hōnaunau than at Kauhakō (Fig 6; χ^2^ > 27.1, df = 3, P < 0.001).

### Prediction c: Probability of reading signs

**Fig 7:**
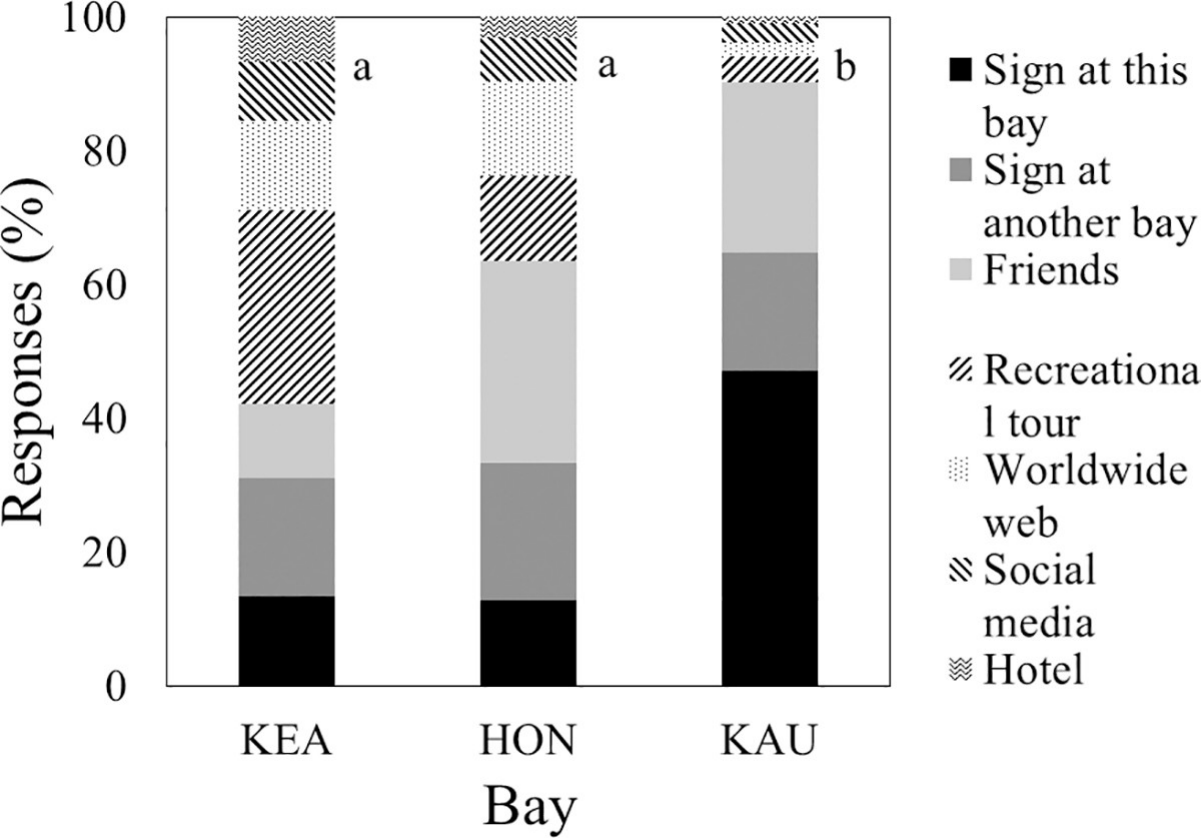
Source of information on existing law limiting interactions of people with dolphins. Sample sizes varied: Kealakekua = 45, Hōnaunau = 63, Kauhakō = 102. Different letters by bars indicate significantly different distributions of information sources.

Respondents who were aware of laws limiting interactions of humans with dolphins were not informed by the same distribution of sources at all bays (χ^2^ = 56.6, df = 12, P < 0.001; Table 1: Q14). Source distributions at Kealakekua and Hōnaunau differed from those at Kauhakō (Fig 7; χ^2^ = 45.3, df = 6, P < 0.001). At Kauhakō, more than two times more visitors learned from signs located at any bay and nearly four times more visitors learned from signs at the bay where they completed the questionnaire than visitors at the other bays. Twelve respondents reported learning from signs at bays other than the bay where they completed their questionnaire. In all cases, these signs were located at one of the other two bays that we surveyed (sample sizes: Kauhakō 5, Kealakekuaʻs south end where kayaks launch for Ka’awaloa 4, Hōnaunau 3). Visitors at all bays reported a similar (H = 0.2, df = 2, P = 0.89) and high probability (mean agreement score = 4.5 or midway between “agree” and “strongly agree”, SE = 0.03) of reading a sign about a local natural resource, if they encountered the sign at a park (Table 1: Q7).

### Prediction d: Compliance with law

**Fig 8:**
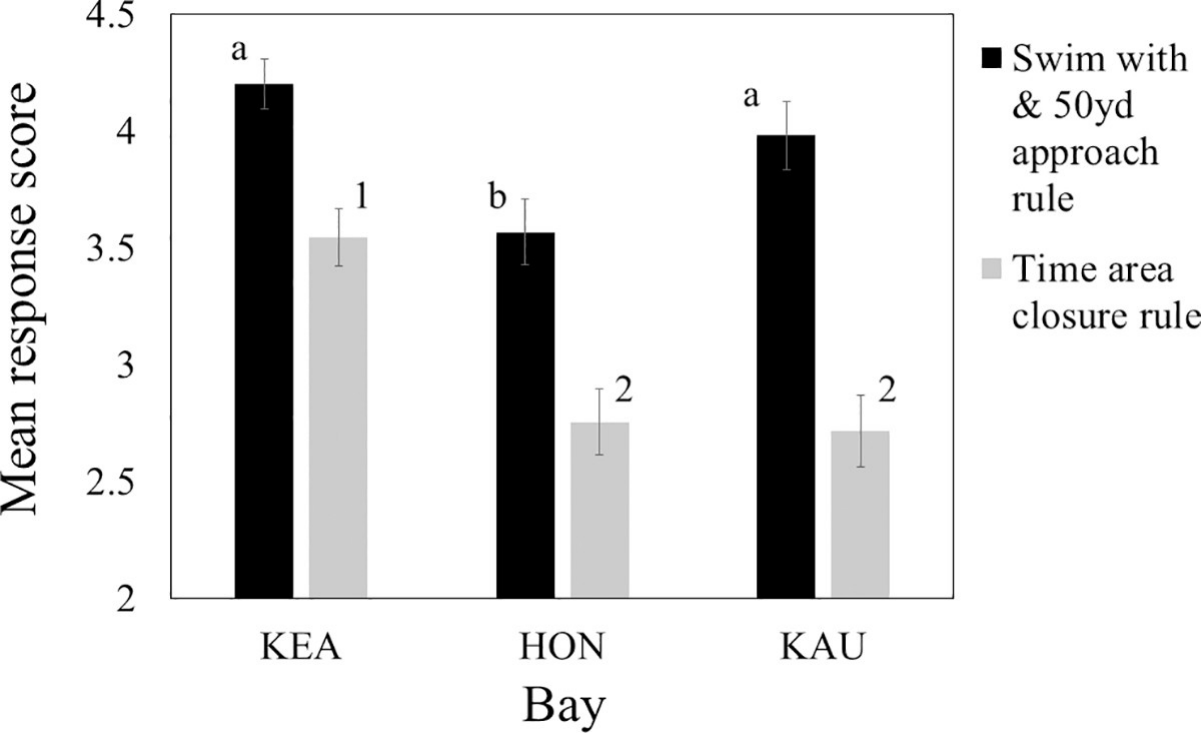
Degree of agreement with potential new rules. Sample sizes varied for swim with and approach rule / time area closure rule: Kealakekua = 110 / 112, Hōnaunau = 120 / 122, Kauhakō = 100 / 98. We translated responses of “strongly disagree”, “disagree”, “unsure”, “agree”, and “strongly agree” to response scores of 1, 2, 3, 4, and 5, respectively. Different letters or numbers by bars indicate significantly different responses to the no swim with and 50yd approach rule or the time area closure rule, respectively. Error bars depict 95% confidence intervals.

Visitors at Hōnaunau reported less agreement with a swim-with and 50yd approach rule than those at Kealakekua and Kauhakō (Fig 8; H = 16.9, df = 2, P < 0.001; for both pairs |Z| > 3.1, P < 0.005; Table 1: Q15). However, all bays had a mean response between “unsure”/neutral and “strongly agree” for this rule. Visitors at Kealakekua reported more agreement with a time area closure rule than those at Hōnaunau and Kauhakō (Fig 8; H = 27.6, df = 2, P < 0.001; for both pairs |Z| > 4.4, P < 0.001; Table 1: Q16). However, all bays had a mean response between “disagree” and “agree” for this rule.

## Discussion

### Bay effect

The three bays we studied attracted people with consistent differences. For every analyzed demographic characteristic of visitors except gender, sample percentage or mean increased from north to south in the study area (Table 2 and Fig 1). This gradient was not surprising given inherent differences among the bays (e.g., accessibility, amenities, and live coral cover) and likely positive correlation of several of the visitor characteristics. For example, Kauhakō was easy to access by car, offered amenities such as sand, legal camping, and concessions, but was less frequented by tourists than the other bays due to the lower live coral cover and longer distance from hotels. These traits probably made it more attractive to frequent visitors, who were often and almost necessarily older, current state residents, and US citizens. Because visitor type varied consistently among bays (prediction e), we followed our original plan for statistical analysis of responses to questions about visitor knowledge and opinion. That is, we began analysis of responses to each analyzed question by testing for a bay effect.

### Test of the research hypothesis

We hypothesized that more obvious and detailed educational signage would reduce the rate at which spinner dolphins would be disturbed by visitors at three bays on the west coast of Hawai‘i Island. To facilitate testing, we generated four specific predictions (a-d) about questionnaire responses that were consistent with this research hypothesis. The first of these related to activities in which visitors planned to engage at the bay. While these activities were diverse and their distribution varied among bays, snorkeling and its positive correlates (watching animals, swimming, and viewing scenery) were consistently popular (Fig 3). As a group, they were the most common planned activities at two bays and the second common planned activities at one bay. Visitors who planned to watch animals, also indicated that they were interested in viewing dolphins, sea turtles, and fish and more interested in viewing them than the other animals in the list of choices (Fig 4). Given these trends, we conclude that dolphins played an important role in visit planning and that prediction a was supported. Assuming that the visitors who wanted to see dolphins would approach the animals if given the opportunity, we felt it was useful to understand what visitors knew about laws limiting approach/interaction.

Knowledge of laws limiting these interactions was low at Kealakekua and Hōnaunau and moderate at Kauhakō (Fig 5). Of those respondents who knew of the law, one fifth were not sure what interactions it prohibited and the remainder could not distinguish interactions that the law prohibited from those that the Dolphin SMART Program discouraged. At the time of our survey, law prohibited harassment, feeding, capturing, and killing of dolphins (i.e., take) and classified harassment as level A (injury) or level B (disturbance, or disruption of behavioral pattern). Dolphin SMART discouraged behaviors that were *likely* to generate disturbance by training and certifying tour operators on safe practices. Although dolphins probably benefit from muddling of the law and Dolphin SMART guidelines, the confusion is only one relatively benign manifestation of the problem with current regulation of interactions of humans with dolphins in Hawai‘i; it is often difficult to know when proximity to a dolphin generates behavioral change and, thus, constitutes disturbance and take under the Marine Mammal Protection Act.

Much of the concern about impacts of recreation on spinner dolphins in Hawai‘i is predicated on dolphin habitat selection, especially preference for protected bays for daytime rest. When bay visitors were asked to identify why dolphins prefer these bays, 47–66% of their responses at each bay were incorrect, with visitors at Kealakekua and Hōnaunau performing worse than those at Kauhakō (Fig 6). Even considering that we removed one “correct” explanation for dolphin preference from final analysis because its selection was correlated with another more common choice, visitor knowledge was fairly poor. Given these trends, we conclude that visitors were not well informed about extant law and its justification, that prediction b was met, and that Kauhakō visitors were more knowledgeable than others. The last is probably explained by that bay’s relatively high rate of visitation by Hawai‘i residents (i.e., more time on island) and/or relatively obvious and detailed signage.

While we cannot control for an effect of high visitor residency without drastically reducing statistical power, we feel that residency’s influence on knowledge was—at most—equal to that of signage for two reasons. First, despite intermediate resident visitation (Table 2), Hōnaunau had visitors with little knowledge of law and its biological rationale (Figs 6 and 7). This level of knowledge was similar to that at relatively poorly-signed Kealakekua and lower than that at relatively well-signed Kauhakō. Second, visitors at Hōnaunau and Kealakekua who knew of law limiting interactions of people with dolphins were nearly four times less likely to have learned about the law from signs at the visited bay (Fig 7). These two patterns and the fact that visitors at all bays were likely to read any educational signs encountered at parks led us to conclude that visitors read obvious and detailed educational signs and that prediction c was supported. Because acquisition of knowledge of law from effective signage need not translate to compliance, we also explored opinions of bay visitors on NOAA’s proposed rule change.

Of the two dolphin interaction rules proposed by NOAA and mentioned on the questionnaire, visitors showed considerable agreement with the swim-with and 50yd approach rule, a bit less agreement with this rule at Hōnaunau than the other bays, and more agreement with this rule than the time area closures (Fig 8). Assuming that at least moderate agreement with a known policy will generate at least moderate compliance, it seemed likely that most visitors would follow known rules on dolphin interactions and that prediction d was partially met. Because predictions a, b, and c were met and prediction d was partially met, we conclude that obvious and detailed educational signs reduce disturbance of and help conserve spinner dolphins in Hawai‘i. This conclusion applies to most of the west side of Hawai‘i Island and other locations where spinners rest during the day, spinners rest in bays, bays are directly accessible by land, and unguided visitors are abundant. Some Hawaiian sites may fail to meet the first criterion (e.g., west coast of Maui, [41]), the second criterion (e.g., west coast of Maui, [41]), or the third criterion (Makako Bay on Hawai‘i Island). Therefore, the utility of signs will vary in a predictable manner across the Hawaiian archipelago.

### Potential limitations from study methods

**Fig 9:**
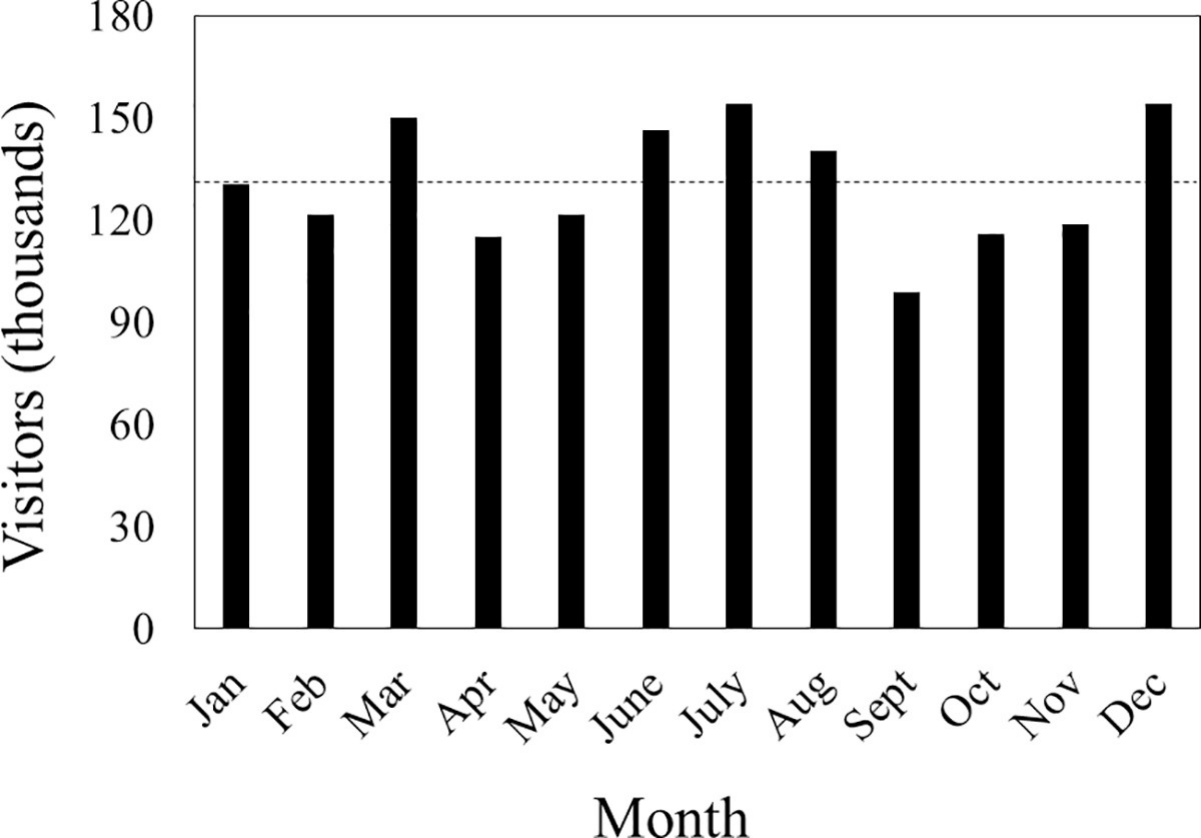
Rate of visitation to Hawai‘i Island’s west coast in 2019. Monthly totals include domestic and international visitors registered with the Hawai‘i Tourism Authority [46]. The dashed horizontal line indicates the monthly mean (130,613 visitors).

The conclusion that we draw from our study could be limited by sampling bias, social desirability bias [42], and/or inference of future behavior from intent and agreement with policy [43, 44]. Sampling bias was unlikely to arise from surveying in May, because resident visitation to bays was probably constant across the year and tourist visitation to Hawai‘i Island’s west coast was only slightly below its monthly average in that month (Fig 9). Responses to questions about visitor demographic characteristics, however, suggested potential bias in favor of females and against native Hawaiians. Still, our limited assessments of functional questionnaire rejection rate by gender and visitation rate by gender indicated that our survey reflected real female-biased visitation to the bays. Underrepresentation of Hawaiians could be explained by disproportionately low bay visitation rates and/or survey methods. For the bays with easy access (Hōnaunau and Kauhakō), we suspect the latter and that this subpopulation tended to visit after we completed our surveying each day, typical daily work hours were over, and most tourists departed for the day. Even if Hawaiians were rare in the sample due to methods, we feel their underrepresentation affects management recommendations and not our conclusion about our research hypothesis for two reasons. Our daily survey period included what is widely known to be the numerical peak in human visitation at these bays and usually included 71% of the typical daily resting period of dolphins (360min) [45].

Questionnaire responses also showed potential signs of social desirability bias. Visitors at all bays tended to be most interested in seeing dolphins/turtles/fish, to believe all described negative interactions of humans with dolphins were illegal, and to report that they read encountered educational signs. Due to the widespread growth in wildlife tourism, we feel the nearly uniform interest in large vertebrates reflected reality. While the other two patterns were more likely to reflect bias, they were each logically combined with results from one or two related question that varied by bay. Thus, both knowledge of regulations and propensity to read signs were inferred from multiple response patterns and unlikely to have been strongly influenced by social desirability bias.

Lastly, we inferred future behavior from both reported likelihood of respondents reading encountered signs and level of agreement with regulations. Because such linkage is tenuous, we qualified our assessment of the single prediction that relied entirely on this type of linkage (i.e., prediction d was “partially” supported). We also suggest (below) that future studies of our topic attempt to quantify the association of intent or opinion with future action.

### Management recommendations

- We interacted with 439 land-based visitors at dolphin resting bays by visiting one of three bays each day over the course of 7 days. Any reasonable extrapolation from this number to the total number of land-based visitors at the three bays per week yields a very great potential for disturbance of resting dolphins by one segment of bay visitors. According to our social survey, this sizeable potential for disturbance could be reduced with improved educational signage. These signs should be installed and maintained at sites meeting the criteria noted at the end of the discussion and follow best practice principles [29]. The latter include (1) posting in a number, size, and location that gets the attention of most approaching visitors [27, 36] and (2) logical linkage of the regulation to its biological rationale [30, 31]. While the type of sign found only at Kauhakō Bay (Fig 2) met several more of the best practice principles than that found at Kealakekua and Hōnaunau Bays [26], new signs should meet as many of the best practice principles as possible, including large and well-spaced text. NOAA should develop and make these signs available for use because it is responsible for implementing the Marine Mammal Protection Act with respect to dolphins and has a staff with considerable expertise in biology, policy, and education. That agency should also install and maintain these signs at sites on resting bays where community organizations are not active, lack funds, and/or lack jurisdiction.
- We propose adding improved educational signage to the policy mix designed to conserve spinner dolphins. This addition need not replace or significantly impair the effectiveness of the other instruments in the mix (e.g., increases in enforcement or education of guided visitors on boats) because signs would primarily compete with other instruments through resource allocation and signs are likely to require far less resources than the other instruments [35, 36]. Instead, the addition may help establish the intensive and multi-faceted management that is required of intensive resource use [47].
- Out of respect to native Hawaiians who still live on and near the focal bays and because our survey may not have captured most Hawaiian visitors at the bays, NOAA should consult Hawaiians regarding at least the content, style, and placement of signs. This process could take the form of a survey tailored to and distributed by Hawaiians and/or formal communications with community organizations.
- If a government agency or community organization opts to add or improve educational signage to help resolve conflict between recreation and wildlife conservation, we urge it to conduct or support experimental tests of the impact of educational signage on human behavior [e.g., 31]. Such studies would help us understand when and to what extent educational signs are effective conservation tools.

## Supporting information

S1 File Questionnaire. (DOCX)

S2 File Analyzed data. (XLSX)
