# Supplementary material for: Better educational signage could reduce disturbance of resting dolphins
Source: PLoS One. 2021 Apr 2;16(4):e0248732. doi: 10.1371/journal.pone.0248732 (PMC8018672; doi:10.1371/journal.pone.0248732)
Supplement: S1 File — (DOCX) [file pone.0248732.s001.docx]

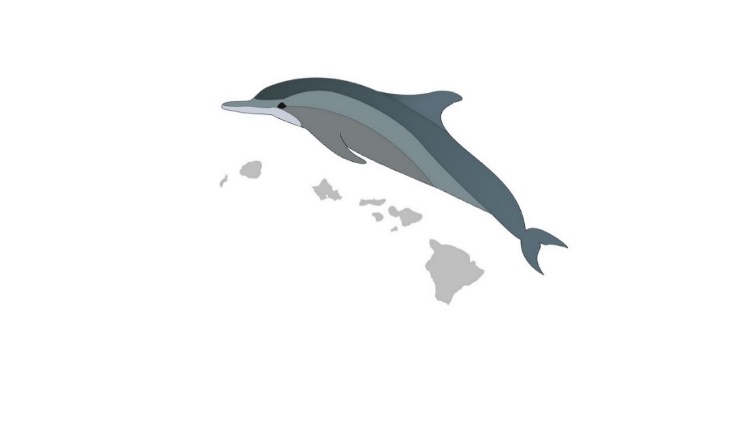


A questionnaire on

Interactions of people with wild spinner dolphins

Distributed with permission from

County of Hawai’i’s Department of Parks and Recreation

Kama’aina United to Protect the ‘Aina / Friends of Ho’okena Beach Park

Keoua Hōnaunau Canoe Club

Oglethorpe University’s Institutional Review Board

State of Hawai’i’s Division of Aquatic Resources

Principal Investigator

Roarke Donnelly

Professor of Biology & Director of Environmental Studies

Oglethorpe University, Atlanta, GA, USA

dolphin@oglethorpe.edu

Rationale for this survey

People and wild spinner dolphins often interact during the day in bays on Hawai’i’s West (Kona) Coast. While these interactions are often enjoyable for people, they can be harmful to dolphins. To encourage interactions that are enjoyable for people and safe for dolphins, we must understand what you know and how you feel about these interactions. This questionnaire was designed to provide this understanding. Mahalo for your participation.

Requirement for survey participation

Respondents must be at least 16yrs old.

Confidentiality policy

Any personal information that you provide will only be used to put responses into groups for statistical analysis or to send you results.

Questions on today’s visit to this bay

1. What did you plan to do while visiting this bay? Please check all options that apply.

__ Camp __ Fish __ Get a tan

__ Kayak __ Rest __ SCUBA dive

__ Snorkel __ Swim __ View scenery

__ Watch animals __ Other:

*If you did not select “Watching animals” in question 1, please skip question 2.

2. When you planned today’s visit to this bay, how interested were you in seeing each of the animal types in the table below?

3. How did you learn about the recreational activities that this bay offers? Please check all options that apply.

__ Family and/or friends __ Hotel or other lodging company

__ Past experience here __ Recreational tour company

__ Social media (specify): __ Website (specify):

__ Other (specify):

*If you received this questionnaire at the south end of Kealakekua Bay, answer question 4 and skip question 5. Otherwise, please skip question 4.

4. How will/did you cover the last 25yards (equals 75feet & 23meters) to your ultimate destination in the bay (that is, where you want/wanted to spend the most time)?

__ Kayak (rented: Yes or No) __ Paddleboard (rented: Yes or No)

__ Swim __ Other (specify):

5. How did/will you cover the last 25yards (equal 75feet & 23meters) to this bay’s shore on your first approach?

__ Hike/walk __ Kayak (rented: Yes or No)

__ Paddleboard (rented: Yes or No) __ Swim

__ Other (specify):

Questions on knowledge and opinions

6. To what extent do you agree or disagree with the following statement? *The conservation of biodiversity is very important.*

(Strongly agree) (Agree) (Disagree) (Strongly disagree) (Unsure)

7. To what extent do you agree or disagree with the following statement? *If I see a sign in a park that has information on a local natural resource, then I read the sign.*

(Strongly agree) (Agree) (Disagree) (Strongly disagree) (Unsure)

*If your answer to question 7 was “Disagree”, “Strongly disagree”, or “Unsure”, please skip question 8.

8. Assume the park sign described in the last question recommends ways to conserve the local natural resource. Which attributes of the sign increase the chance that you will follow the recommendations? Please check all of the first five options that apply or the last option.

__ Content is consistent with more detailed material on a referenced, government website

__ Content is consistent with more detailed material on a referenced, non-profit website

__ Identifies a trusted authority that endorsed the sign’s content

__ Identifies a trusted authority that provided the sign’s funding

__ Lists some of the scientific publications supporting the sign’s content

__ None of the above attributes increase the likelihood

9. Assume you are interested in learning about a local natural resource and have access to the information in several formats. If all formats were offered at the same moment and required the same amount of your time, how would you rank the formats? Use ranks 1-4, with 1 indicating the most preferred format.

__ Narrated animation on the web

__ Printed Brochure

__ Text and images on the web

__ Video interview of an expert on the web

10. Spinner dolphins frequently visit this bay during the day. What attracts them to this location? Please check all options that apply.

__ Calm water for rest __ Food provided by humans

__ Natural sources of food __ Safety from predators

__ Social interactions with humans __ Unsure

__ Other (specify):

11. Is human interaction with spinner dolphins in this bay currently limited by law?

__ Yes __ No __ Unsure

*If you answered “No” or “Unsure” to question 11, please skip questions 12 through 14.

12. What level of government created and enforces the current law limiting human interaction with spinner dolphins?

__ City __ County __ State __ Federal __ Unsure

13. What interactions of humans with spinner dolphins are currently illegal within 2 nautical miles of shore on main Hawaiian Islands? Please check all options that apply.

__ Capturing a dolphin

__ Disrupting a dolphin’s behavioral pattern

__ Intentionally moving within 50yards (equals 150feet or 46meters) of a dolphin while swimming

__ Intentionally moving within 50yards of a dolphin by watercraft with motor in gear

__ Unsure

14. From where have you learned about existing law limiting interactions of humans with spinner dolphins? Please check all options that apply.

__ Friends and/or family

__ Hotel or other lodging company

__ Recreational tour company

__ Sign at other bay (specify):

__ Sign at this bay

__ Social media (specify):

__ Website (specify):

__ Other (specify):

15. To what extent would you agree or disagree with a law—if it existed—that attempted to protect Hawaiian spinner dolphins by making it illegal for humans to intentionally move by any means within 50yards (equals 150feet & 46meters) of dolphins?

(Strongly agree) (Agree) (Disagree) (Strongly disagree) (Unsure)

16. To what extent would you agree or disagree with a law—if it existed—that attempted to protect Hawaiian spinner dolphins by closing portions of three West Hawai’i bays to people from 6am-3pm each day?

(Strongly agree) (Agree) (Disagree) (Strongly disagree) (Unsure)

17. Rate each of the following entities according to how much you trust it to communicate or endorse accurate information on the natural resources of Hawai’i’s West (Kona) Coast. Please use a scale of 1 through 4, with 1 indicating the most trust and U indicating “Unsure”.

1 2 3 4 U 🡪 County of Hawai’i’s Dept. of Parks & Recreation

1 2 3 4 U 🡪 Kama’aina United to Protect the ‘Aina / Friends of

Ho’okena Beach Park

1 2 3 4 U 🡪 National Oceanic & Atmospheric Administration

1 2 3 4 U 🡪 State of Hawai’i’s Division of Aquatic Resources

1 2 3 4 U 🡪 State of Hawai’i’s Division of State Parks

Questions on personal attributes

18. Including your current visit, how many times have you visited this bay in the last year? ____

19. If you would like to receive a summary of findings from this survey, please clearly print your e-mail address below.

20. Are you a U.S. citizen?

__Yes __No; I am a citizen of____________________

*If you answered “No” to question 20, please skip question 21.

21. Are you a resident of the state of Hawaii? That is, do you spend the majority of each year in Hawaii at property that is owned by you or your immediate family?

__ Yes (for ___ years) __ No

22. Do you consider yourself to be native Hawaiian by blood/ancestry?

__ Yes __ No __ Unsure

23. What is your age in years? ___

24. What is your gender?

__ Female __ Male __ Transgender __ Other (specify):

25. Please share your comments on interactions of humans with wild spinner dolphins, conservation policy, and/or this survey.
